# Efficacy of pulsed-xenon ultraviolet light on reduction of *Mycobacterium fortuitum*

**DOI:** 10.1177/2050312120962372

**Published:** 2020-10-10

**Authors:** Thomas W Huber, Emma Brackens, Piyali Chatterjee, Frank C Villamaria, Lauren E Sisco, Marjory D Williams, John David Coppin, Hosoon Choi, Chetan Jinadatha

**Affiliations:** 1 Research Service, Central Texas Veterans Health Care System, Temple, TX, USA; 2 Department of Internal Medicine, Baylor Scott & White Memorial Hospital, Temple, TX; 3 Medical Service, Central Texas Veterans Health Care System, Temple, TX, USA; 4 Department of Medicine, College of Medicine Texas A&M University, Bryan, TX, USA

**Keywords:** Ultraviolet disinfection, pulsed-xenon ultraviolet, *Mycobacterium fortuitum*

## Abstract

**Objectives:**

Hospitals and healthcare facilities rely largely on isolation and environmental disinfection to prevent transmission of pathogens. The use of no-touch technology is an accepted practice for environmental decontamination in medical care facilities, but little has been published about the effect of ultraviolet light generated by a portable pulsed-xenon device use on Mycobacteria. We used *Mycobacterium fortuitum* which is more resistant to ultraviolet radiation and less virulent than *Mycobacterium tuberculosis*, to determine the effectiveness of portable pulsed-xenon devices on *Mycobacterium* in a laboratory environment.

**Methods:**

To determine the effectiveness of pulsed-xenon devices, we measured the bactericidal effect of pulsed-xenon devices on *Mycobacterium fortuitum.*

**Results:**

In five separate experiments irradiating an average of 10^6^ organisms, the mean (standard deviation) log-kill at 5 min was 3.98 (0.60), at 10 min was 4.96 (0.42), and at 15 min was 5.64 (0.52).

**Conclusions:**

Our results demonstrate that using pulsed-xenon devices is a highly effective modality to reduce microbial counts with this relatively ultraviolet germicidal irradiation–resistant mycobacterium in a time-dependent manner.

## Introduction

Ultraviolet germicidal irradiation (UVGI) can cause sufficient damage to the deoxy-ribonucleic acid (DNA) and cellular structures of microorganisms so as to render them incapable of replication.^1,2^ Multiple trials have demonstrated the effectiveness of ultraviolet (UV) light devices in reducing the environmental bioburden of pathogenic organisms such as methicillin-resistant *Staphylococcus aureus* (MRSA), vancomycin-resistant *Enterococcus* spp (VRE), *Clostridioides difficile* (C. diff), *Acinetobacter spp.*, and norovirus.^3–6^ Furthermore, many studies have shown that UVGI produced from a portable pulsed-xenon UV emitting device (PPX-UV) used in conjunction with manual disinfection of the patients environment reduces the risk of healthcare-acquired infections (HAI).^2,7^

Transmission of mycobacterial infections is not uncommon in healthcare settings.^8^ Infections from Mycobacterium can be related to environmental exposure of patients and healthcare workers to these organisms.^9^ UV-based air system purification has been previously used and found to be effective.^9^ Besides the primary airborne route of transmission from person to person, the bacilli can survive for prolonged periods outside the body on surfaces if they are protected from direct sunlight, that is, in dark areas. Inadequate environmental cleaning is an additional risk for HAI, and the use of PPX-UV can help to mitigate this risk. Bacilli residing on surfaces can be transferred through multiple routes including equipment (bronchoscope or an endotracheal intubation and suctioning with mechanical ventilation) and/or supplies that can introduce the organism into the respiratory tract. The susceptibility of mycobacteria to UVGI varies with the species tested.^10–12^ Since *Mycobacterium tuberculosis* (MTB) is a highly pathogenic organism it was thought best to conduct a feasibility study on a similar organism before conducting the study on the more biologically dangerous and pathogenic organism, MTB.

*Mycobacterium fortuitum* has been shown to be more resistant to UV than its more pathogenic cousin MTB.

Most information concerning the susceptibility of *M. fortuitum* to UVGI has been done using mercury vapor lamps which emit continuous UVGI with a narrow emission spectrum centered at 254 nm. *M. fortuitum* is naturally more resistant to mercury generated UVGI than non-mycobacteria and also more resistant than other mycobacteria. Lee et al.^13^ found that 20 mJ/cm^2^ of mercury generated UV light resulted in more than 3 log reduction of *Mycobacterium avium, Mycobacterium intracellulare*, and *Mycobacterium lentiflavum* but 50 mJ/cm^2^ were required for a 3 log reduction of *M. fortuitum*. The irradiation Ct value for a 3 log inactivation of *M. fortuitum* was 600 times higher than for *Escherichia coli*. Other mycobacteria were only 2–10 times more resistant to UV killing than *E. coli*. The effectiveness of different UV technologies on MTB have been tested previously both in a hospital setting and in the environment.^14–17^ Pulsed UV, a new UV device that delivers high-intensity bursts of energy in short time periods and thereby, better penetration than the commonly used mercury generated UVGI sources. By examining the impact of PPX-UV on *M. fortuitum*, a species that is more resistant to UVGI than MTB, insight can be gained on the potential for PPX-UV technology which emits high-intensity pulsed light ranging from 200 to 315 nm to reduce the level of MTB contamination in clinical environments.

## Methods

The experiments were approved by the Research & Development Committee and conducted at Central Texas Veterans Health Care System, Temple, TX over a 11-month period in a BSL-3 facility. The study was designed as laboratory based with quantitative analysis. The effect of UV exposure for the survival of *M. fortuitum* was quantified as log reduction of colony forming unit of *M. fortuitum*. Strain type of *M. fortuitum* (ATCC 6841) was purchased from ATCC. On day 1, a 7-day culture of *M. fortuitum* on Lowenstein Jensen medium was inoculated to Middlebrook 7H9 broth containing 6–8 sterile 3 mm glass beads to disperse clumps. On days 2–5, the 7H9 broth culture was incubated and vortexed daily for 20 s. On day 7, the turbidity of the 7H9 broth culture was adjusted to a MacFarland 1 density standard. Serial 10-fold dilutions of 10^−1^ through 10^−6^ of the standardized suspension were prepared in sterile 7H9 broth containing glass beads. We modified the bead mixing method of Kent and Kubica;^18^ after each suspension or dilution was prepared it was vortexed for 20 s and allowed to settle for 10 min before transfer. Dilution or inoculum transfers were aspirated from the top of the solution to minimize transfer of large clumps of mycobacteria.

Duplicate 100 mm petri dishes containing 7H11 media were inoculated with 0.1 mL of 10^−3^ through 10^−6^ dilutions of 7H9 broth suspensions. Colonies were enumerated after 5–7 days in a 35°C incubator containing 5% CO_2_. Triplicate 150 mm plates containing 7H11 agar was inoculated with 0.1 mL of the MacFarland 1 suspension. Inoculum was spread to avoid small scratches in the media which would allow organisms to evade irradiation and form colonies to prevent blocking of UV rays by the sides of the petri dish, or shielding of organisms between the media edges and the petri dish. Uncovered plates were placed on a rack in the front of a biological safety cabinet (BSC) at a 45° angle from horizontal, a height of 0.83 m, 4 feet from the PPX-UV device. This position prevented the glass front of the BSC from blocking UV rays. The plates were irradiated for 5, 10, and 15 min. The lids were replaced and the plates incubated at 35°C in a dark CO_2_ incubator. Surviving colonies were enumerated after 5–7 days of incubation. The experiment was repeated on five different days. The log-kill was calculated by: Log survivors − Log inoculum = Log-kill.

### Statistical analysis

A Bayesian multilevel linear regression model was used to determine the mean log-kill at each timepoint, while partially pooling the data across the experiments. Log-kill was modeled as a function of time and included a varying intercept for the experiment. Results are expressed as the model estimated mean log-kill and 95% uncertainty interval at each time point. The raw data are plotted and color coded by experiment. The Bayesian model was run in the “brms” package in R, which uses the Bayesian inference software Stan. Plots were created using “ggplot2” package in R version 3.6.3.

## Results

**Figure 1.**
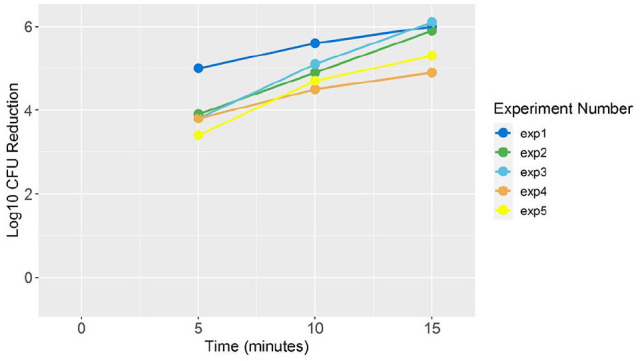
Log-kill of *Mycobacterium fortuitum* at 5, 10, and 15 min irradiation using Xenon-generated ultraviolet light at 4 ft distance from the source.

The log-kills of *M. fortuitum* (ATCC 6841) at 5, 10, and 15 min of PPX-UV exposure are shown graphically in Figure 1. The mean (SD) log-kill at 5 min was 3.98 (0.60), at 10 min was 4.96 (0.42), and at 15 min was 5.64 (0.52). The model estimated mean log-kill partially pooled across the experiments was 4.03 (3.36–4.68) at 5 min, 4.86 (4.25–5.50) at 10 min, and 5.69 (5.04–6.39) at 15 min.

## Discussion

Prior studies have shown mycobacteria to be more resistant to traditional UVGI than other bacteria such as *E. coli*.^10,12,13^ In contrast, our results using PPX-UV showed a 10-min irradiation kill rate of 5 logs for *M. fortuitum*; similar to Hosein’s study using PPX-UV against multi-drug resistant organisms like MRSA and VRE.^6^ This demonstrates the efficacy of PPX-UV against *M. fortuitum* and potentially MTB. We attribute the higher kill rate at 5 min in our study to facilitate killing of single or very small aggregates of organisms on the surface of the growth medium by our modified bead mixing method.^18^ Larger aggregates of organisms likely take more UV exposure to be inactivated but would still form a colony if there was only one survivor in the aggregate. The slowing of the kill rate between 10 and 15 min may be due to inaccessibility of organisms in aggregates or moisture from the media rather than resistance to killing by irradiation. Similarly, our modified bead mixing method resulted in more organisms in higher dilution plate counts than lower dilutions. Bacterial aggregates usually cause problems in assessing susceptibility to irradiation since the depth of UV penetration for polymers is about 25 µm. Organisms on the bottom of a medium to large clump or a thin film would likely be protected from irradiation and thus form a colony.^19^ This could also be correlated to the presence of organic material in a real hospital room in the absence of manual cleaning. More studies are needed to confirm our initial findings.

This study adds to the existing literature on the use of portable UV devices for surface disinfection in the hospital setting.^2,13,18^ *M. fortuitum* serves as a logical surrogate for MTB due to its decreased virulence and increased resistance to UVGI and potentially validates the use of PPX-UV for disinfection of surfaces in rooms occupied by patients with MTB.

Our study has limitations. The experimental conditions used in this study does not fully reproduce the UV disinfection of *M. fortuitum* in a clinical setting. The use of UV device for germicidal purpose becomes less effective if the distance between the target and the UV source increases. In addition, the UV dose which depends on the intensity and the duration functions best at a shorter distance and when in direct line of the source. Therefore, the objects in proximity to the light source would need shorter disinfection cycles compared to objects further away. Shadowed areas would require longer disinfection cycles. Type of material also affects the efficacy of UV radiation, in particular a few organic materials are known to have poor reflection rates and UV can penetrate them. The experiments designed in this manuscript to test the efficacy of the PPX-UV on *M. fortuitum* were performed at a shorter distance and in direct line of the UV source.

## Conclusion

Our study demonstrates that PPX-UV device can act as an effective bactericidal source for *M. fortuitum.* Furthermore, the germicidal activity of PPX-UV increases in a time-dependent manner. This study is significant because portable UVGI devices are becoming commonly used for area disinfection in hospital settings.
